# News and Notes

**Published:** 1907-09

**Authors:** 


### NEWS AND NOTES.

A movement is on foot to establish a large city hopital in Charlotte.

Three hundred cases of dengue fever have been reported at Brownsville, Texas.

According to a report there are between 5,000 and 6,000 cases of tuberculosis in Baltimore.

Ninth French Congress for Internal Medicine will be held at Paris, October 17th-19th, 1907.

Mr. E. S. Wilcox, Kauai, has given $50,000 for the establishments of a hosital for children at Honolulu.

Ten cases of yellow fever were reported August 10th, from the military post of Cienfuegos, Cuba.

The Connecticut Legislature has voted to allow $40,000 for the tuberculosis work of the Hartford Hospital.

An epidemic of typhoid is said to exist at Tuskegee Normal School, where 27 cases, with 2 deaths, have been reported.

Dr. T. O. Powell, superintendent of the Georgia Sanatarium at Milledgeville, died of pneumonia at Tate Springs, Tenn., August 18th, 1907.

The Medical Department of the University of Manila will be opened in September. It is proposed to devote special attention to tropical medicine.

The Department of Agriculture of the State of Pennsylvania has recently discovered that a certain brand of patent food is composed of ground corncobs.

---

The Atlanta School of Medicine has opened its course of study to women and it is reported that quite a number are to avail themselves of this opportunity.

---

The Journal A. M. A. states that the total number of medical students in the United States for the year ending June 30th, 1907, was 24,276, a decrease of 928 below 1906 and a decrease of 1,871 below 1905.

---

Dr. Sir William Henry Broadbent, physician in ordinary to the King and Prince of Wales and consulting physician to St. Mary's Hospital, Paddington, and other London hospitals, died on July 10th, at the age of 72 years.

---

Dr. David G. Austin, Lainsburg, Michigan, who, it is claimed, sold his practice to Dr. George Robb, Flushing, and agreed not to practice in Laingsburg, has been enjoined from practicing in that place on complaint of Dr. Robb.

---

The Paris Medical Faculty has recently announced that henceforth the incumbents of the special chairs of anatomy, histology, physics, chemistry, and pharmacology will not be allowed to take positions as physicians or surgeons in the hospitals.

---

The General Committee of the Cancer Research Fund of London, referring to the alleged cures for cancer, reported that no curative value could be attacked to any of those which had been tested and that trypsin had shown no effect on the growth and development of tumors.

---

The reverse side of the transfers used on the various surface lines of New York, contain, on certain days, instructions to consumptives and the addresses of the various tuberculosis dispensaries. The privilege is a courtesy from the business firm which usually advertises there.

According to the figures of the bureau of statistics the number of beds available in the public hospitals and other charitable institutions in Austria is 37,610, or one bed for ech 700 of the population.

---

The following rare dislocation of the tibia is referred to in a recent issue of Puck, under the head of Fashion, which is said "leads a woman to willingly cut her sleeves off above the elbow and then hustle to find $5.27 to buy cotton, silk or kid gloves to cover the end of her tibia."

---

Dr. J. T. Killibrew, a prominent young physician of Mobile, Ala., was killed in a railway automobile accident July 24th. Dr. Killibrew was president of the Mobile Medical Society, lecturer on diseases of women in the University of Alabama, and an assistant in the Ingo-Bonduarant Infirmary.

---

The state board of health at its semi-annual meeting held at its offices in the capitol, decided to ask the legislature for an appropriation of approximately $12,000 for the purpose of establishing a plant for the manufacture of diphtheria anti-toxin, to be furnished free in all cases where it is needed for the treatment of patients.

---

The Fifth District Medical Society of the Association of Georgia met in Douglasville, July 17th, and after an interesting programme the following officers were elected for the ensunig year: President. Dr. T. R. Whitley, Douglasville: vice-president, Dr. E. G. Jones, Atlanta; secretary and treasurer, Dr. J. Ross Simpson, Atlanta.

---

At its meeting last year the German Association of Medical Editors decided to prepare a list of authors, who are accustomed to furnish, for pay, articles recommending new remedies and the preparations of chemical manufactures. Their articles are neither to be published nor abstracted in the journals of the members of the association.

